# Removal of Antibiotics from Water by Polymer of Intrinsic Microporosity: Isotherms, Kinetics, Thermodynamics, and Adsorption Mechanism

**DOI:** 10.1038/s41598-020-57616-4

**Published:** 2020-01-21

**Authors:** Mohammed N. Alnajrani, Omar A. Alsager

**Affiliations:** National Center for Irradiation Technology, Nuclear Science Research Institute, King Abdulaziz City for Science and Technology, P.O. Box 6086, Riyadh, 11442 Saudi Arabia

**Keywords:** Analytical chemistry, Environmental chemistry, Pollution remediation

## Abstract

Traces of antibiotics within domestic and industrial effluents have toxic impact on human health as well as surrounding flora and fauna. Potential increase in antibiotic resistance of microorganisms is likely to rise due to the incomplete removal of antibiotics by traditional wastewater processing, methods such as membrane filtration and biological treatment. In this study, we investigated a novel class of material termed Polymer of Intrinsic Microporosity (PIM) that is based on amorphous microporous organic materials for the application of antibiotic removal form aqueous environments. The adsorption of four commonly used antibiotics (doxycycline, ciprofloxacin, penicillin G, and amoxicillin) was evaluated and found that at least 80% of the initial concentrations was eliminated under the optimized conditions. Langmuir and Freundlich models were then employed to correlate the equilibria data; the Freundlich model fit well the data in all cases. For kinetic data, pseudo-first and second order models were examined. Pseudo-second order model fit well the kinetic data and allowed the calculation of the adsorption rate constants. Thermodynamic parameters were obtained by conducting the adsorption studies at varied reaction temperatures. Surface potential, adsorption at various solution pHs, thermogravimetric analysis (TGA), Infrared spectroscopy (IR), and surface area experiments were conducted to draw possible adsorption mechanisms. The removal of antibiotics from water by PIM-1 is likely to be governed by both surface and pore-filling adsorption and could be facilitated by electrostatic interactions between the aromatic rings and charged functional groups as well as hydrogen bond formation between the adsorbent and adsorbate. Our work shows that the application of such novel microporous material could contribute to the removal of such challenging and persistent contaminants from wastewater with further optimizations of large-scale adsorption processes.

## Introduction

Antibiotics are chemical compounds with a wide spectrum of applications in humans and veterinary medicine^1^. They are used for treatment of diseases caused by various bacterial infections in addition to their wide usage in animal farming and aquaculture activity for disease prevention and growth promotion purposes^2–4^. For example, the yearly consumption of antibiotics was estimated to be 162,000 tons for China^5^, 13,000 tons for United Sates^5^, and, 10,000 tons for European countries^5^. Significant portions of the administered doses (30–90%) are excreted unmetabolized as active forms^6,7^. These active antibiotic residues are found in the environment (surface water, ground water, and soil) as a result of runoff of domestic, agricultural, and industrial effluents^8^. Some of the commonly used antibiotics were found to be persistent with long half-lives. Thus, they potentially pose adverse effects to water quality and aquatic life^9,10^. Widespread use of antibiotics alters microbial ecosystems and exerts selective pressure on susceptible bacteria and lead to the survival of resistant strains and development of antimicrobial resistance, making existing antibiotics ineffective in curing various newly emerging infectious diseases^11–14^. Besides the long term impact, antibiotics could trigger allergic reactions in certain individuals and disrupt the native microbial system when they access the human body via the food chain and drinking water^5,15^.

Conventional water and wastewater treatment technologies based on biological treatment, filtration, coagulation, flocculation, and sedimentation have been found to be ineffective to completely remove antibiotics and can only achieve partial elimination^16–18^. Advanced oxidation technologies that produce ^•^OH radicals to actively and non-selectively decompose contaminants, such as UV photolysis, photo-catalysis (H_2_O_2_ and ozone), Fenton’s reagent, and ionizing radiation, were found to achieve complete removal of antibiotics^19–23^. However, main constraints of these methods are related to application cost, catalyst management and residual toxicity in treated effluents and resulting byproducts^24,25^.

Such issues have motivated active research in recent years to develop new alternative technologies that are simple and more efficient in eliminating antibiotics from the bodies of water. The adsorption approach provides various advantages compared to other treatment technologies, such as low initial investment, simpler construction (reactor/absorber) design, easy operation, and ability to be tailored to be selective or non-selective in nature^26,27^. Various adsorbents have been successfully developed for the removal of antibiotics from aqueous environments, including multiwall carbon nanotubes^28^, activated carbons^29^, zeolites^30^, β-cyclodextrin polymer^31^, clay^32^, and metal organic frameworks^33^. Amongst these adsorbents, zeolite and activated carbon are the most studied materials due to their microporous (pore size <2 nm) and mesoporous (pore size 2–50 nm) properties, making them highly active and capable of antibiotic removal^34–37^. The development of adsorbent that can mimic zeolite and activated carbon is a vibrant research area covering a wide range of organic and inorganic hybrid materials^38,39^.

Recently, a novel class of polymeric materials emerged where microporosity is intrinsic to their molecular structures. Such a class is known as Polymer of Intrinsic Microporosity (PIM) which has exhibited analogous behavior to that of crystalline and amorphous microporous and mesoporous materials in a number of applications including gas separation, adsorption, and catalysis^40–43^. PIM possesses unusual structural features with a backbone composed of fused rings and site of contortion resulting in high free volume as they cannot pack space efficiently^44^. Investigations of this materials showed high surface area, high thermal and chemical stability, and potential application in adsorption as it is soluble in common organic solvents and can be readily processed in various application-driven forms including powders, membranes and fibers^45–47^. Although a number of PIMs have been successfully synthesized, the first generation polymer designated as PIM-1 (poly(1- trimethylsilyl-1-propyne) (PTMSP) and poly(4-methyl-2-pentyne) (PMP)) received considerable attention in the adsorption of materials such as the removal of various dyes^48^ and phenol^49^ from aqueous solutions and removal of aniline from air and aqueous phases^50^. Application of PIM-1 in the removal of emerging organic contaminates such as antibiotics has yet to be explored and demonstrated.

**Figure 1:**
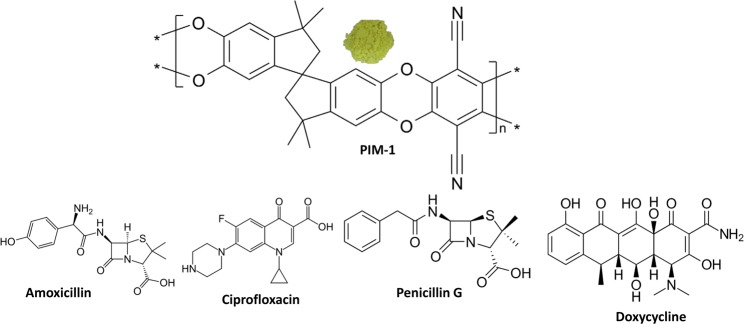
Molecular structure of PIM-1 (only repeating unit) and the studied antibiotics. A photo of PIM-1 powder is shown.

With this background, the objective of this study was to examine the behavior of PIM-1 at different experimental conditions to remove four commonly used antibiotic compounds: doxycycline, ciprofloxacin, penicillin G, and amoxicillin; from aqueous solutions in an agitated batch regime. Figure 1 shows molecular structures of the studied antibiotics and PIM-1. Adsorption isotherms, kinetics, thermodynamics were investigated and important parameters were determined. Optimum operation conditions were determined for future large-scale adsorption process.

## Experimental

### Materials

Dimethylformamide, dimethylacetamide, acetonitrile, toluene, methanol, chloroform, acetone, and deuterated chloroform were purchased from MERCK and were used as received. Tetrafluoroterephthalonitrile (TFTPN, 98%, MERCK) was heated to around 150 °C and the pure product collected without vacuum. 5,5′,6,6′-Tetrahydroxy-3,3,3′,3′-tetramethyl-1,1′-spirobisindane (TTSBI, 98%, Alfa Aesar) was dissolved in methanol and re-precipitated from dichloromethane before use. Anhydrous potassium carbonate (K_2_CO_3_, 99.0%, MERCK) was dried in an oven at 110 °C overnight before use. Amoxicillin, doxycycline, ciprofloxacin, and penicillin G were purchased from Alfa Aesar. Double distilled water (Mill-Q with a conductivity of 18.2 MΩ.cm) was used to make stock solutions and the desired concentrations of the studied antibiotics.

### Synthesis of PIM-1

Previously reported PIM-1 synthesis protocol was followed^45,50^. The protocol yielded 51 g with a 90% conversion. GPC: *M*_n_ = 1.0 × 10^5^ g.mol^−1^, *M*_w_ = 1.9 × 10^5^ g.mol^−1^, PDI = 1.90. ^1^H-NMR (400 MHz, CDCl_3_): 6.79 (br, s, 2 H), 6.39 (br, s, 2 H), 2.30 (br, s, 2 H), 2.13 (br, s, 2 H), 1.34–1.28 (br, 12 H). IR (cm^−1^): 2957, 2867, 2244, 1608, 1450, 1266.

### Batch adsorption experiments

#### Adsorption kinetics

Batch adsorption experiments for the four different antibiotics were conducted with an initial concentration of 200 µM. Five mL solution of the target antibiotics was mixed and stirred (using magnetic bar at a speed of 400 rpm) with PIM-1 (2 mg) at different adsorption times, pH, and temperatures. The solution’s pH was adjust using diluted sodium hydroxide or hydrochloric acid solutions. The samples were filtered with 0.22 µm syringe-filters from Millipore prior to UV-VIS measurements. Unknown concentrations were determined by a comparison against calibration curves. Calibration curves were established from four different concentrations (50, 100, 150, and 200 µM) with excellent linearity in all cases (R^2^ > 0.999). UV-VIS spectra were recorded before and after adsorption using LAMBDA 850 UV/Vis spectrophotometer from PerkinElmer. Quartz cuvettes from PerkinElmer were used for the measurements. The reported adsorption data points in this study represent the average values of three individual experiments with standard deviations not exceeding 5%.

#### Adsorption isotherms

Five mL solutions of the target antibiotics (neutral pH) with different concentrations (50, 100, 150, and 200 µM) were mixed and stirred (using magnetic bar at a speed of 400 rpm) at room temperature with the adsorbent PIM-1 (2 mg) at different adsorption times specific for each antibiotic. Equilibrium times were 24 hours for amoxicillin and penicillin-G and 5 hours for ciprofloxacin and doxycycline. Determination of equilibrium was experimentally achieved by measuring the adsorbate concentration and determining the saturation point. Antibiotic concentrations were measured as described above. Adsorption capacity of PIM-1, Q_e_, was calculated according to Eq. 1^51^:

1 $${{\rm{Q}}}_{{\rm{e}}}=\frac{({{\rm{C}}}_{0}-{{\rm{C}}}_{{\rm{t}}}){\rm{V}}}{{\rm{m}}}$$

where Q_e_ is the adsorption capacity at equilibrium (mg g^−1^); C_0_ and C_e_ represent the initial and equilibrium concentrations of antibiotics (mgL^−1^), respectively; V is the volume of antibiotic solutions (5 mL); and m is the mass of the adsorbent PIM-1 (mg).

### Characterizations

PIM-1 molecular weight was measured by an Agilent gel-permeation chromatograph (GPC) equipped with a ZORBAX PSM 300-S column. The instrument was calibrated with standard polystyrene samples. THF was used as an eluent and was run at a flow rate of 1 mg mL^−1^. The thermogravimetric analysis (TGA) was carried out using a Perkin Elmer TGA7 with a temperature range from 25 °C up to 800 °C (with an elevation rate of 10 °C min^−1^) under nitrogen constant flow. ^1^H-NMR spectra were recorded at room temperature using JOEL 600 MHz and polymer solutions were prepared in CDCl_3_. Infrared spectroscopy (PERKIN ELMER16F PC FT-IR) instrument equipped with an attenuated total reflectance accessory was used to characterize PIM-1 and PIM-1-adsorbed antibiotics. Each sample was scanned 16 times at a resolution of 4 cm^−1^. Water content was removed from the adsorbent through filtration and overnight drying at 40 °C. Brunauer–Emmet–Teller (BET) surface area and pore parameter measurements were conducted using Quantachrome instrument (Nova touch LX^2^ model) for 50 mg of PIM-1 samples and 200 µM concentration of the target antibiotics. The samples were left under vacuum for four hours at 120 °C before the analysis. BET surface area was determined by using a multi-point method, while pore parameters were calculated by Dubinin-Astakhov (DA) method. Surface potential measurements were conducted using a Microtrac instrument (Zeta-check model) at 20 °C. Five mL of PIM-1 suspensions with various experimental conditions (different antibiotics or in different pH values) were used for the measurements.

## Results and Discussion

### Synthesis and characterization of PIM-1

PIM-1 is synthesized from the reaction of 5,5′,6,6′ tetrahydroxy-3,3,3′,3′ tetramethylspirobisindane with 1,4 dicyanotetrafluoro benzene. This reaction can be carried out by two different methods: high temperature method (at 155 °C)^45,52^ or low temperature method (at 65 °C)^41,46^. In this study, PIM-1 was produced following the high temperature approach, due to the fact that it can be obtained in a shorter time, and the produced PIM-1 was fully characterized. The appearance of as prepared PIM-1 shows significant fluorescent yellow color. The produced PIM-1 has a molecular weight of 1.9 × 10^5^ g mol^−1^ and a polydispersity index of 1.90 (as detailed in the experimental section). IR and ^1^H-NMR (detailed in the experimental section) measurements indicated that the prepared PIM-1 was pure and similar to those previously reported^45,50,52^. The characteristic nitrile (CN) stretches at 2240 cm^−1^ with the aromatic and aliphatic C-H stretches around 3000 cm^−1^ were observed. Additionally, the presence of six different proton environments in ^1^H NMR spectrum was displayed (more details are mentioned in the experimental section). Thermal stability of PIM-1 was confirmed by conducting TGA experiment where the polymer backbone degradation was observed at 450 °C, indicating a thermally stable adsorbent material.

Furthermore, the nitrogen gas adsorption and desorption isotherms with their pore size distribution of PIM-1 were studied. The observed isotherm can be characterized as a type II according to the IUPAC classification^53^. BET specific surface area was found to be 630 m^2^ g^−1^ and the total pore volume was 0.514 cm^3^ g^−1^. Pore size distribution, based on density functional theory, shows characteristics of microporosity (<2 nm). These data are comparable to the previously reported figures of PIM-1^45,50,52^. The lack of efficient backing of the solid state macromolecules forming PIM-1 arises from its contorted shape and extreme rigidity. Degradation of the porous structure as a result of bond rotation is prohibited, due to the presence of spirocenters and the rigid fused-ring structure^43,44^. Having confirmed these remarkable chemical and physical properties of PIM-1, we proceeded to apply this potential adsorbent to remove antibiotics from aqueous solutions.

### Adsorption isotherms

An adsorption isotherm characterizes the distribution of the adsorbate molecules between the liquid phases at various equilibrium concentrations^54^. Important insights into the adsorption process, such as how the interaction between adsorbent and adsorbate occurs, are obtained by finding the suitable model that fits well the adsorption data. The most popular adsorption models are Langmuir^55^ and Freundlich^56^. These two models were employed in their linear forms^57,58^, shown in Eqs 2 and 3:

2 $$\frac{{{C}}_{{e}}}{{{Q}}_{{e}}}=\frac{1}{{{Q}}_{{m}}{{K}}_{{L}}}+\frac{{{C}}_{{e}}}{{{Q}}_{{m}}}$$

3 $${Ln}\,{{Q}}_{{e}}=\,{Ln}\,{{K}}_{{\boldsymbol{F}}}+\frac{1}{{n}}\,{Ln}\,{{C}}_{{e}}$$

where C_e_ (mg L^−1^) is the equilibrium concentration of antibiotics, Q_e_ (mg g^−1^) is the amount of antibiotics adsorbed per gram of adsorbent PIM-1 under equilibrium, Q_m_ (mg g^−1^) is the theoretical maximum adsorption capacity of PIM-1 for antibiotics, and K_L_ (L mg^−1^) is a constant describing the affinity in the process of Langmuir adsorption; K_F_ is the Freundlich empirical constant reflecting the relative adsorption capacity of the PIM-1, and 1/n is a constant indicative of the intensity of the Freundlich adsorption^57,58^.

**Figure 2:**
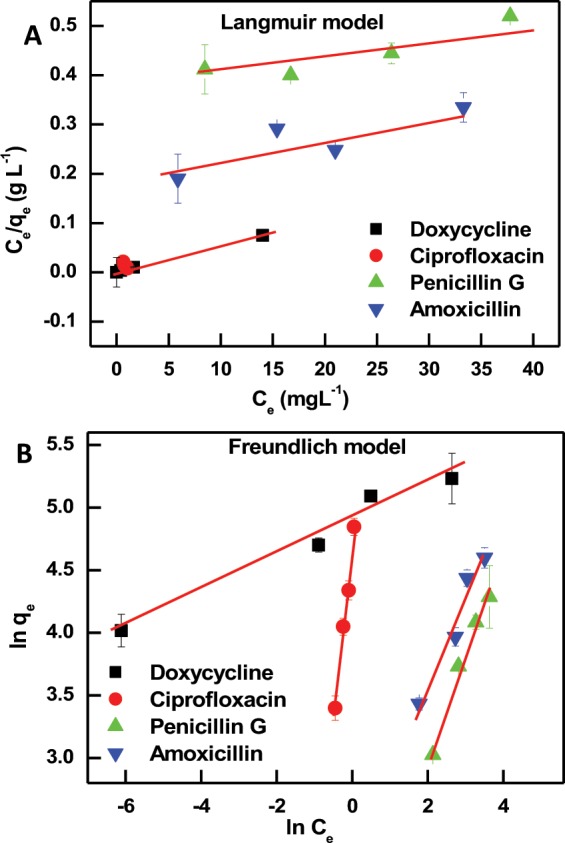
Langmuir (**A**) and Freundlich (**B**) adsorption isotherms of the adsorption data of the studied antibiotics onto PIM-1. The range of ciprofloxacin data in Langmuir fit (**A**, x-axis: 0.6–1 and y-axis: 0.008–1) is smaller than the data range of rest of the studied compounds and appears as a single data point in the figure. Error bars represent the standard deviation of the mean of three experiments.

As shown in Fig. 2, the adsorption data of the studied antibiotics onto PIM-1 were fitted with both the Langmuir and Freundlich isotherms. They are known as two-parameter models, which can provide information on the adsorption capacity and constants related to the adsorption affinity. The Langmuir model assumes that the adsorption takes place in a homogeneous surface (sites of equal accessibility and energy) resulting in the formation of a monolayer of adsorbate on the surface of the material that saturates the pores and prevents the transmigration. The Freundlich model assumes that adsorption is not ideal and reversible, occurring through formation of multilayers of adsorbate on a non-uniform heterogeneous surface^57,59^.

From the data fits shown in Fig. 2 and the correlation coefficient values (R^2^) presented in Table 1, the Freundlich model seems to be the best fit for the adsorption process of penicillin G and amoxicillin. Doxycycline and ciprofloxacin are almost equally fitted with the Langmuir and Freundlich models. Characterizing the adsorption to be chemisorption (monolayer) or physisorption (multilayer) is not only dependent on the adsorbent material, but also on the adsorbate. Fitting the isothermal data alone would not result in firm conclusions regarding the nature of the adsorption^60^. However, with the data presented later in this study (Section 3.5), it can be concluded that penicillin G and amoxicillin are adsorbed only by physisorption mechanism (as Table 1 shows very poor fitting for chemisorption mechanism). On the other hand, doxycycline and ciprofloxacin are well-described by both adsorption mechanisms. Previous studies have shown that both adsorption mechanisms could coexist in the same adsorption system^58,61^. Thus, the variations encountered in modeling the adsorption of the studied antibiotics is attributed to their chemical nature and their affinity for adsorption onto PIM-1. According to the Freundlich model, the values of the constant 1/n were less than 1 (Table 1) and indicated favorable adsorption^60^.

**Table 1:** Parameters (average values with a standard deviation not exceeding 5%) of Langmuir and Freundlich isotherm models for antibiotic uptake onto PIM-1.

Compound	Model	K_L_ or K_F_	Q_m_ (mg g^−1^) or 1/n	R^2^
Doxycycline	Langumir	5.3	189	0.999
	Freundlich	134.3	0.144	0.975
Ciprofloxacin	Langumir	0.76	33.10	0.94
	Freundlich	106.0	0.28	0.99
Penicillin G	Langumir	0.01	257	0.83
	Freundlich	3.5	0.85	0.98
Amoxicillin	Langumir	0.03	213	0.88
	Freundlich	8.75	0.70	0.95

The highest adsorption capacity (Q_m_) for the studied antibiotics is reported for penicillin G followed by amoxicillin, which were found to follow the physisorption mechanism with multilayer adsorption. These two compounds belong to the same antimicrobial family (β-Lactam) and differ only from one another by two functional groups (amine and hydroxyl), their chemical structures are presented in Fig. 1. The modeling of their uptakes resulted in similar isothermal characteristics in both Langmuir and Freundlich models, shown in Table 1. Ciprofloxacin resulted in the lowest Q_m_ value (33.10 mg g^−1^) which could be attributed to the limited number of functional groups compared to the other studied compounds. Generally, the reported Q_m_ values are better or comparable to other adsorbent materials previously reported for the removal of the same classes of the studied compounds. For example, the value of Q_m_ was found to be 47 mg g^−1^ for amoxicillin, 315 mg g^-1^ for penicillin G, and 44.4 mg g^−1^ for tetracycline when bentonite, activated carbon, and mesoporous silica were used, respectively^62–64^.

Furthermore, the favorable nature of adsorption can be expressed in terms of the dimensionless separation factor R_L_^65^ for an adsorbate that obeys the Langmuir model^60^. It can be calculated using the Langmuir constant K_L_ presented in Table 1, according to R_L_ = 1/(1 + K_L_C_0_)^60^. R_L_ values indicate the following: irreversible isotherm (R_L_ = 0), favorable (0 < R_L_ < 1), linear (R_L_ = 1) or unfavorable (R_L_ > 1). The dimensionless separation factors calculated for doxycycline and ciprofloxacin are 0.002 and 0.019, respectively. R_L_ values were less than 1 and greater than zero indicating favorable adsorption.

### Adsorption kinetics

**Figure 3:**
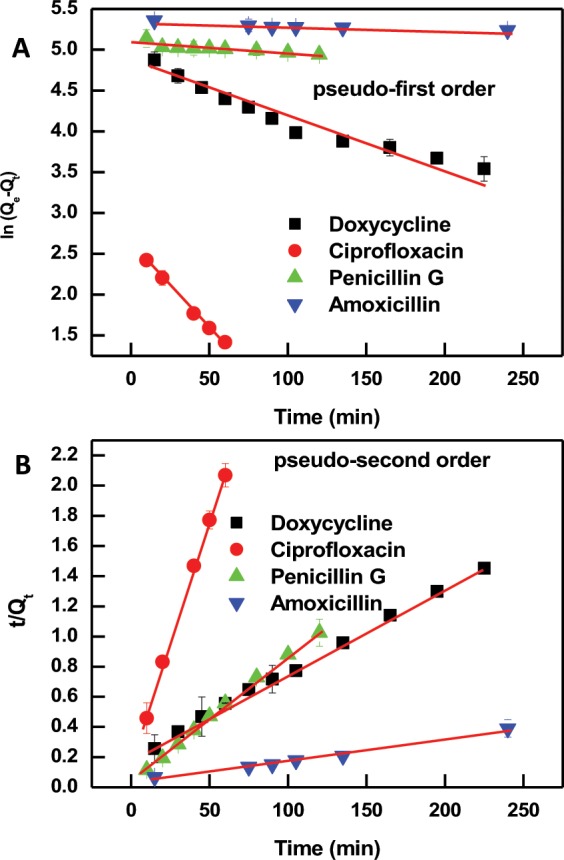
Pseudo-first order (**A**) and pseudo-second order (**B**) kinetic model analysis of the adsorption data of the studied antibiotics onto PIM-1. Error bars represent the standard deviation of the mean of three experiments.

Designing an effective and sustainable adsorption system requires deeper understanding of the dynamic of the reaction in terms of orders of the rate constants^66^. Therefore, adsorption kinetics data for the studied antibiotic systems were analyzed (as shown in Fig. 3) by two different kinetic models using pseudo-first order^67^ and pseudo-second order^68^ using their linear forms^60^:

4 $$Ln({Q}_{e}-{Q}_{t})=Ln\,{Q}_{e}-{K}_{1}t$$

5 $$\frac{t}{{Q}_{t}}=\frac{1}{{K}_{2}{Q}_{e}^{2}}+\frac{t}{{Q}_{e}}$$

where Q_e_ and Q_t_ denote the adsorption capacity of antibiotics at the equilibrium state and at time of t, respectively; K_1_ (min^−1^) and K_2_ (g mg^−1^ min^−1^) are the modulus of pseudo-first-order and pseudo-second-order adsorption, respectively^60^.

Table 2 shows the parameters obtained from the fits of the adsorption kinetic models. The correlation coefficients (R^2^) of the pseudo-second order model for all the studied antibiotics were greater than 0.99, with the exception of amoxicillin (R^2^ = 0.88) indicating the applicability of this fitting model. The implication of this finding is that the adsorption of antibiotics by PIM-1 is likely to be kinetically controlled as a second-order reaction and the adsorption is dependent on the concentration of both adsorbent and adsorbate. According to the pseudo-second-order rate constant, the fastest adsorbing compound is ciprofloxacin followed by amoxicillin, penicillin-G, and doxycycline; respectively. The reported rate constants are broadly consistent with those reported for the same antibiotics^59,61,69,70^. The pseudo-second order kinetic model suggests that chemical adsorption is the dominant mechanism, involving electrostatic attraction, which is consistent with the variable pH experiments presented in the coming section.

**Table 2:** Parameters (average values with a standard deviation not exceeding 5%) of the adsorption kinetic models.

Compound	Pseudo-kinetic- order	K_1_ (min^−1^) or K_2_ (g mg^−1^ min^−1^)	Q_e_ (mg g^−1^)	R^2^
Doxycycline	First-order	0.0061	121.2	0.94
	Second-order	0.00015	178.60	0.99
Ciprofloxacin	First-order	0.0203	13.60	0.99
	Second-order	0.0062	31.15	0.99
Penicillin G	First-order	0.0013	161.95	0.75
	Second-order	0.00018	191.04	0.99
Amoxicillin	First-order	0.0005	27.17	0.78
	Second-order	0.00023	208.60	0.88

Additionally, the trend of adsorption capacity at equilibrium predicted by the pseudo-second-order model (listed in Table 2) is consistent with the trend of maximum adsorption capacity found by Langumir model, listed in Table 1. Both pseudo-second-order model and Langumir model found that the highest adsorption capacity is four β-Lactam antibiotics followed by doxycycline, and the least adsorption capacity is for ciprofloxacin. It should be noted that the limits of solubility of the studied antibiotics in aqueous solvents are higher than the highest target concentration (200 µM) used herein^71–73^. Thus, aggregation and precipitation do not contribute to any of the observed results.

### BET surface area, IR and TGA characterizations of antibiotic adsorption

**Figure 4:**
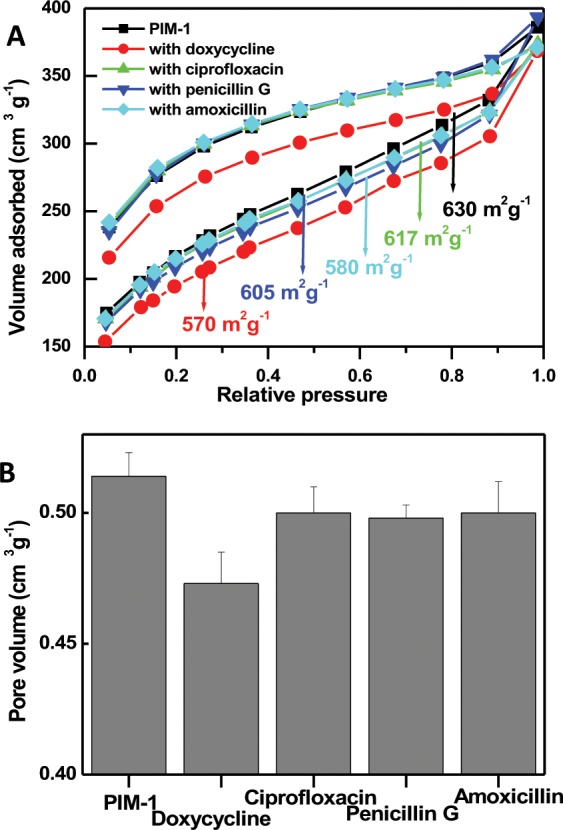
BET surface area (**A**) and pore volume (**B**) for PIM-1 powder before and after the adsorption of the studied antibiotics. Error bars represent the standard deviation of the mean of three experiments.

The large surface area of PIM-1 promotes it as a promising candidate for adsorption of antibiotics from aqueous environments. BET surface area measurements showed that PIM-1 powder has an apparent surface area of about 630 m^2^ g^−1^. It shows a type II isotherm at low pressure indicating significant microporosity. A hysteresis behavior was observed upon N_2_ desorption that could be due to polymer swelling, as shown in Fig. 4. It is well known that the surface area of PIM-1 is strongly influenced by the processing history^74^. Treatment of PIM-1 with methanol for 48 hours resulted in higher surfer area (775 m^2^ g^−1^)^75^.

Detailed N_2_ adsorption/desorption isotherms and pore parameters data are provided in Fig. 4. The large surface area determined by N_2_ gas adsorption is due to the adsorption of the gas molecules not only to the surface of PIM-1, but also inside the pores. BET surface area measurements were repeated for PIM-1 after adsorption of the studied antibiotics when the adsorption process was terminated at equilibrium (specific for each antibiotic, as detailed in Sections 2.3 and 2.4).

As shown in Fig. 4A, different degrees of surface area reduction were recorded upon adsorbing the investigated antibiotics and modeling the N_2_ gas adsorption/desorption. The values of surface area are inserted in Fig. 4A. Active sites responsible for N_2_ gas adsorption were saturated by the presence of adsorbed antibiotics. It could be assumed that the pore filling adsorption takes place due to the moderately surface area reduction due to antibiotics adsorption. For example, there is approximately 60 m^2^ g^−1^ surface area reduction when doxycycline was adsorbed. It is difficult to assume that the adsorption process occurs only on the surface while PIM-1 pores are freely accessible by the small-sized antibiotic molecules, smaller than the PIM-1 pore size^59,69^. Evidently, Fig. 4B shows that there were different degrees of pore volume reduction of PIM-1 after the adsorption of antibiotics. Furthermore, the isothermal data fitted by Freundlich model supports the heterogeneous nature of adsorption with uneven distribution of adsorbed molecules over the active sites, which suggests pore filling adsorption. Similar conclusions were reported with the adsorption of phenol molecules by PIM-1^49^.

**Figure 5:**
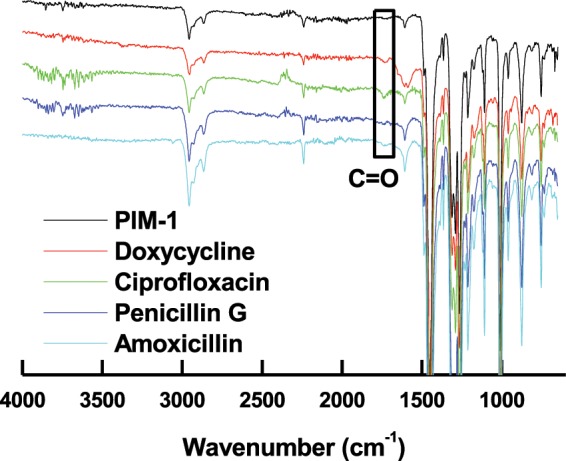
IR spectra of pure PIM-1 powder and after adsorption of the studied antibiotics. The appearance of the carbonyl group (C=O) in the spectra of adsorbed target molecules is highlighted.

Further investigation of antibiotic adsorption was conducted by IR spectroscopy. As shown in Fig. 5, a typical PIM-1 IR spectrum is shown with the characteristic nitrile (CN) stretches at 2240 cm^−1^ and the aromatic and aliphatic C-H stretches around 3000 cm^−1^ ^48^. The IR spectrum for PIM-1 with adsorbed antibiotics resulted in the appearance of a new peak at ~1740 cm^−1^, which is attributed to a carbonyl group (C = O) present in the molecule structures of adsorbed antibiotics, shown in Fig. 1. No other peaks identifying the presence of the antibiotics could be recorded due to 1) lack of sensitivity of IR spectroscopy to proportionally small adsorbed antibiotic quantities, and 2) shielding effect of PIM-1 polymeric porous structure to antibiotic signals.

**Figure 6:**
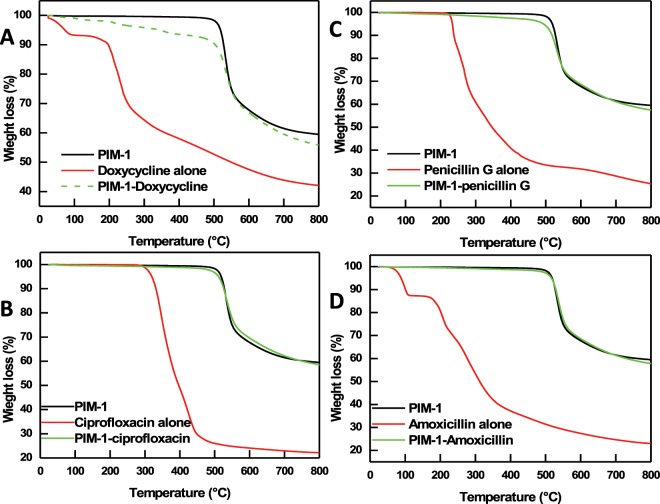
TGA analysis of thermal decomposition of PIM-1 before and after the adsorption of doxycycline (**A**), ciprofloxacin (**B**), penicillin G (**C**), and amoxicillin (**D**). The thermal decomposition of studied antibiotics alone is also presented.

The data obtained by BET surface area and IR spectroscopy are further supported by TGA measurements. Figure 6 shows a TGA curve of powder PIM-1 sample. Consistent with previous reports^50^, PIM-1 is stable up to 460 °C, after which polymer backbone degradation occurs. TGA experiments were repeated for PIM-1 powder after the adsorption of antibiotics, shown in Fig. 6A–D. Around 2–8% weight loss is observed for PIM-1 loaded with antibiotics below 400 °C, which is due to the decomposition of the adsorbed antibiotics, as their decomposition was found to sharply occur between 100–300 °C (shown in Fig. 6A–D). The gradual thermal decomposition behavior of the adsorbed antibiotics compared to the sharper decomposition of the free forms suggests encapsulation within PIM-1 pores that provide protection against thermal decomposition. TGA results are in line with the abovementioned BET surface area and IR data.

### Effect of experimental conditions: insights into the adsorption mechanism

#### Effect of solution pH

**Figure 7:**
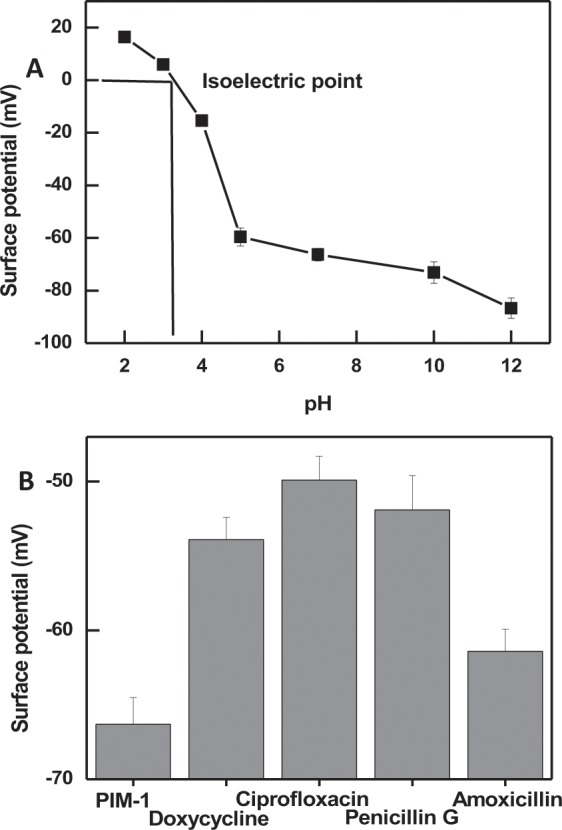
(**A**) Shows the surface potential measurements for PIM-1 under increasing pH values for the determination of the isoelectric point and (**B**) shows the surface potential measurements of PIM-1 following the adsorption of each antibiotic at pH 7. Error bars represent the standard deviation of the mean of three experiments.

Solution pH could play a major role in the adsorption process of a molecule, as pH variation could promote changes in the charges of adsorbent and adsorbate^50,60^. In the present study, an important aspect of antibiotic adsorption is the charge distribution on PIM-1 surface under different pH media. Surface potential measurements can provide information about the charge and charge distribution on the surface of PIM-1. Figure 7A shows different surface potential values for PIM-1 polymeric powder suspended in solutions with different pHs values. The isoelectric point (IP) was determined to be 3.3, where PIM-1 carries zero net charge^76^. At pH lower than the IP, PIM-1 is positively charge while solutions with pHs higher than the IP result in a negatively charged PIM-1.

Furthermore, Fig. 7B shows that the adsorption of the studied antibiotics at neutral pH resulted in alternation of the original surface potential of PIM-1. The highest surface potential change was observed with the adsorption of ciprofloxacin and the smallest change associated with the adsorption of amoxicillin. Such variation could be attributed to the fact that the studied antibiotics could present different charges at neutral pH due to the differences in the values of acid dissociation constants (pKa), shown in Table 3. It is worth noting that the major contributor to the resolved surface potential values are the surface charges. This is due to the dependence of phase analysis light scattering technique (used to measure surface potential) on electrophoretic movability influenced by solution-polymer interphase^77^. Thus, surface potential data indicates that the adsorption of antibiotics occurs on the exterior phase of PIM-1 surface; while pore filling adsorption cannot be ruled out and was previously confirmed by the surface area measurements.

**Table 3:** chemical parameters of the studied antibiotics^71,72^.

Antibiotic	Molar mass g. mol^−1^	pk_a_	Log P
Doxycycline	444.43	3.3; 7.7; 9.7	−1.30
Ciprofloxacin	331.347	6.09; 8.74	0.28
Penicillin G	334.39	2.7	1.38
Amoxicillin	365.40	2.7, 7.5, 9.6	0.87

**Figure 8:**
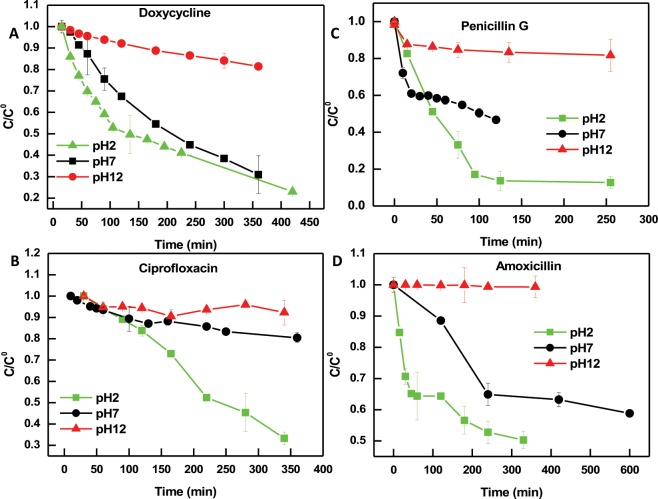
Adsorption studies for doxycycline (**A**), ciprofloxacin (**B**), penicillin G (**C**), and amoxicillin (**D**) at different pH conditions. Error bars represent the standard deviation of the mean of three experiments.

The effect of solution pH on the adsorption of antibiotics onto PIM-1 was further investigated by repeating the kinetic experiments at acidic or basic conditions. As shown in Fig. 8, a common feature across all the investigated antibiotics is that the adsorption was greatly diminished at a solution pH of 12 compared to acidic and neutral conditions. At pH 12, the surface of PIM-1 is highly negatively charged with a surface potential of -86.7 mV. Additionally, all the reported pK_a_ values of the investigated compounds fall below pH 12 which indicate that the functional groups including hydroxyl, amine, and carboxylic acid; that are abundantly available in the molecular structure of the adsorbates; will be deprotonated bearing negative charges^58,69^. Thus, electrostatic repulsion between the negatively charged PIM-1 and the negatively charged adsorbate could be the dominant effect explaining the diminished adsorption activity at pH 12.

Additionally, hydrolysis of nitrile group (CN) and the formation of amide in the polymer backbone was previously reported in basic sodium hydroxide solution; carboxylic acid, ammonium carboxylate, and sodium carboxylate structures were also confirmed^45^. The formation of these functional groups was observed within few hours, which is likely to occur in the batch adsorption studies conducted herein and could additionally explain the decreased adsorption functionality of PIM-1 towards the studied antibiotic at pH 12.

Due to complex chemical nature of the studied antibiotics, it is expected that distinct adsorption behavior for each molecule could be observed at different experimental conditions. As can be seen in Fig. 8, the adsorption of doxycycline slightly prefers the neutral pH compared to acidic pH, although both conditions reached nearly the same removal percentage (75%) if enough adsorption time is provided. On the contrary, ciprofloxacin, Penicillin G, and amoxicillin were adsorbed more effectively at pH 2 compared to other pH solutions. At pH 2, PIM-1 is positively charged with a surface potential of 16.4 mV and this characteristic could increase the hydrophobicity of PIM-1 in acidic solution, which leads to a further enhancement in the adsorption of the moderately hydrophobic antibiotics (seen in Fig. 8). The pK_a_ values of the aliphatic carboxylic acid group of amoxicillin and penicillin G are close to pH 2, as shown in Table 3. Thus, this group is not fully protonated bearing partial negative charges and could facilitate electrostatic attraction between the adsorbent and adsorbate. A common feature of these three compounds is their moderate hydrophobicity with positive log P values, shown in Table 3. Previous studies of PIM-1 surface modification have shown that replacing the nitrile group with more hydrophilic groups (amide, carboxylic acid, ammonium carboxylate and sodium carboxylate) at high pH values increased its solubility in more polar organic solvents^45^. Although this study conducts the experiments in aqueous media and PIM-1 remains insoluble in all experimental conditions, it could be speculated that acidic pH could increase the hydrophobicity of the polymer^58^, which might contribute to the enhanced adsorption of the moderately hydrophobic antibiotics (completely dissolved in water under the current experimental conditions). Similar trends were observed during the adsorption of phenol and its derivatives onto activated carbon^58^.

#### Effect of temperature and thermodynamic parameters

**Figure 9:**
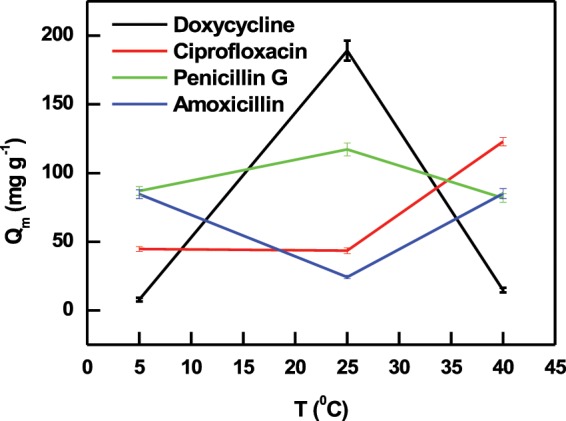
Effect of solution temperature on the adsorption of antibiotics by PIM-1. Error bars represent standard deviation from three experiments.

Effect of adsorption process temperature and determination of thermodynamic parameters were conducted at three different temperatures, i.e. 5, 25 and 40 °C for 3 hours. As shown in Fig. 9, three main observations could be drawn from the behavior of antibiotic adsorption by PIM-1 at increasing adsorption temperature: A) adsorption is favored at room temperature (the case of doxycycline and penicillin G), B) increasing adsorption temperature does not improve antibiotic uptake (the case of amoxicillin), and C) increasing adsorption temperature improved the antibiotic uptake (the case of ciprofloxacin). Such complex behavior highlights the challenging nature of the adsorption of this class of contaminants and the need to accurately optimize the adsorption process if a given compound is being treated. It was hypothesized that specific temperature dependence is associated with given adsorbates as their solubility could be decreased or increased with temperature variation, and therefore influencing their solid phase adsorption^78^. It is worth mentioning that all these three scenarios were previously reported for the adsorption of various organic pollutants by various adsorbents^79^.

The thermodynamic parameters of antibiotic adsorption onto PIM-1, such as Gibbs free energy (ΔG^0^), enthalpy (ΔH^0^) and entropy (ΔS^0^) were determined using the variation of solute distribution coefficient between the solid and liquid phases (K_d_), through the following equations^60,78^:

6 $${{K}}_{{d}}=\frac{{{q}}_{{e}}}{{{C}}_{{e}}}$$

7 $$\Delta {{G}}^{0}=-\,{RT}\,{Ln}(1000\times {{K}}_{{d}})$$

8 $$Ln(1000\times {{K}}_{{d}})=\frac{-{\Delta }{{H}}^{0}}{{RT}}+\frac{{\Delta }{{S}}^{0}}{{R}}$$

where R is the universal gas constant (8.314 J mol^−1^ K^−1^) and T is the absolute temperature of the system (K), and the factor 1000 is multiplied by K_d_ to make it dimensionless^78,80,81^. K_d_ must be a dimensionless parameter since the unit of ΔG^0^, gas constant, and temperature are J mol^−1^, J mol^−1^ K, and K, respectively. Since the adsorption of antibiotics was conducted using aqueous solutions with very low concentration of the target compounds, the dimensionality of the K_d_ (L g^−1^) can be easily converted into dimensionless values by the multiplication of the distribution coefficient by 1000 (as 1 L = 1000 g, and the solution density is 1 g mL^−1^)^78,80,81^. Furthermore, System’s ΔH^0^ and ΔS^0^ values were determined from the slope and intercept of van’t Hoff plot (ln (1000 x K_d_) versus 1/T), respectively. The calculation of ΔG^0^ resulted in negative values in all cases indicating spontaneous adsorption^69,78^, as shown in Table 4. The spontaneity (increase in the negativity of ΔG^0^ values) was increased with the more favorable adsorption temperature, as seen in the case of ciprofloxacin adsorption.

**Table 4:** Thermodynamic parameters (as average values with uncertainty not exceeding 5%) for antibiotics adsorption onto PIM-1.

Compound	Temperature (K)	K_d_ (Dimensionless)	ΔG^0^ (kJ mol^−1^)
Doxycycline	278	140	−11.4
	298	7040	−22.0
	313	500	−16.2
Ciprofloxacin	278	2550	−18.1
	298	2440	−19.3
	313	63470	−28.8
Penicillin G	278	2700	−18.3
	298	5850	−21.5
	313	2400	−20.3
Amoxicillin	278	2150	−17.8
	298	380	−14.7
	313	2170	−20.1

The current study applies the dimensionless distribution coefficient (K_d_) to predict the system’s ΔH^0^ and ΔS^0^, as shown in Eq. 8. Their values were determined from the slope and intercept of van’t Hoff plots (ln (1000 x K_d_) versus 1/T). It was found that the use of the dimensionless K_d_ in our system to calculate ΔH^0^ and ΔS^0^ is not appropriate, resulting in plots with very poor correlation coefficients (R^2^ value of doxycycline is 0.18, ciprofloxacin is 0.36, amoxicillin is 0.003, and penicillin G is 0.001). Similar observation was reported by a previous study of the adsorption of cadmium ions onto orange peel^78^. Other methods for computing ΔH^0^ and ΔS^0^ such as implementing Langmuir and Freundlich constants is being studied and will be reported in a future publication.

## Conclusions

The characteristics and mechanism of four commonly used antibiotics adsorption onto PIM-1 were thoroughly investigated. The batch experimental results showed that the isothermal data are best fitted by the Freundlich model. Kinetic studies revealed that kinetic data were well described by the pseudo-second order model. Surface potential, adsorption at various solution pHs, TGA, IR, and surface area experiments confirmed that the removal of antibiotic from water by PIM-1 is governed by both surface and pore-filling adsorption and could be facilitated by electrostatic interactions between the aromatic rings and charged functional groups between the adsorbent and adsorbate. Evaluation of the Gibbs free energy (ΔG^0^) identified that the adsorption process is spontaneous as the values were negative. Finally, the study showed that PIM-1 is capable of removing a wide range of antibiotics with nearly 80% removal at the optimized conditions from aqueous solutions within short operation time. Our work showed that the application of such novel microporous material could contribute to the removal of such challenging and persistent contaminants from wastewater.
